# Bulk Acoustic Wave Characteristics of Pseudo Lateral-Field-Excitation on LGT Single Crystal for Liquid Phase Sensing

**DOI:** 10.3390/s19051076

**Published:** 2019-03-03

**Authors:** Jiachao Xu, Tingfeng Ma, Liang Yan, Mingfei Wang, Ji Wang, Jianke Du, Chao Zhang

**Affiliations:** 1 School of Mechanical Engineering and Mechanics, Ningbo University, Ningbo 315211, China; xujiachao0251@sina.com (J.X.); mollovey@163.com (L.Y.); wangmingfei_nbu@163.com (M.W.); wangji@nbu.edu.cn (J.W.); dujianke@nbu.edu.cn (J.D.); 2 Research Institute of Tsinghua University in Shenzhen, Shenzhen 518057, China; zhangchao1969@tsinghua-sz.org

**Keywords:** bulk acoustic wave, LGT crystal, lateral-field-excitation, impedance characteristics, liquid phase sensing

## Abstract

In the present study, pseudo lateral-field-excitation (LFE) bulk acoustic wave characteristics on LGT crystals are investigated to increase the sensitivity of LFE devices on the liquid characteristic variations. The cut orientation of LGT crystals for pseudo-LFE is investigated and verified experimentally. For an LFE device in the pseudo-LFE mode, the thickness shear mode wave is excited by the thickness field rather than the lateral field. The present work shows that when the (yxl) 13.8° LGT plate is excited by the electric field parallel to the crystallographic axis *x*, it operates in the pseudo-LFE mode. Moreover, characteristics of devices including the sensitivity and impedance are investigated. The present work shows that sensitivity of LFE devices to variation of the conductivity and permittivity of the aqueous solution are 9 and 3.2 times higher than those for AT-cut quartz crystal based devices, respectively. Furthermore, it has been found that the sensitivity of the LGT LFE sensor to liquid acoustic viscosity variations is 1.4 times higher than the one for the AT-cut quartz sensor. The results are a critical basis of designing high-performance liquid phase sensors by using pseudo-LFE devices.

## 1. Introduction

Bulk acoustic wave devices are widely employed to manufacture acoustic wave sensors. Studies show that lateral-field-excitation (LFE) bulk acoustic waves are sensitive to variation in the electrical and mechanical characteristics of the analytes [1]. Therefore, LFE-based sensors are capable of providing more useful information from analytes in comparison to the conventional thickness-field-excitation (TFE) devices [2,3,4,5,6,7]. Hempel et al. analyzed the AT-cut quartz LFE devices from impedance point of view [8]. They suggested that the contacting electrolyte can alter the electrical field distribution of the LFE device. For an LFE device in the pseudo-LFE mode, the thickness shear mode wave is excited by the thickness field rather than the lateral field.

Studies indicated that with the low piezoelectric coupling factor, the AT-cut quartz LFE device has low sensitivity to variations of the electrical characteristics of the aqueous medium. Therefore, investigation of LFE sensors based on other piezoelectric crystals with remarkable coupling factors, are highly demanded for improving the sensitivity of LFE sensors. The higher piezoelectric coupling factor the device has, the greater changes the resonance parameters of the TSM can be caused by changes of the liquid electrical parameters because of the piezoelectric coupling effect. Thus, a high piezoelectric coupling factor is a suitable feature for an LFE bulk acoustic sensor. Langatate (LGT) single crystals have high piezoelectric coupling factors, good temperature characteristics and very small acoustic losses [9,10]. Therefore, LGT crystals are favorable for applications in bulk acoustic wave sensors. However, the uncertainty in the cut orientation of LGT crystals for pseudo-LFE hindered the investigation of LGT crystals on LFE liquid phase sensors.

In order to find an appropriate cut of LGT for Pseudo-LFE, piezoelectric coupling factors and acoustic wave velocities of LGT crystals are calculated. LFE devices with operating frequency of 5 MHz on LGT are designed and manufactured in this regard. Furthermore, the device impedance properties are investigated to validate the accuracy of the cut. In addition, the device sensitivities on a variety of the aqueous solution conductivities, permittivities, and acoustic viscosities are investigated. The frequency-time response distributions of the LGT LFE devices are illustrated. Furthermore, the results obtained are compared with those of AT-cut quartz LFE devices.

## 2. Theory

Studies show that the piezoelectric coupling factor of an anisotropic crystal depends on the cutting direction and the implemented electrical field orientation [11]. Electrodes can be placed in the resonator surface at any orientation. Therefore, LFE devices are with the higher degree of freedom. This makes the piezoelectric coupling factors of LFE more complicated in comparison to those of TFE.

The extended Christoffel-Bechmann method [12] is performed to predict LFE coupling factors. It is intended to investigate the LFE piezoelectric coupling factors of TSM (i.e., b and c modes) of the (yxl) 13.8° LGT plate. b and c indexes denote the fast and slow thickness shear modes, respectively. Moreover, in order to analyze the pseudo-LFE mode, the coupling factors for the thickness-field-excitation of the (yxl) 13.8° LGT plate are calculated.

**Figure 1:**
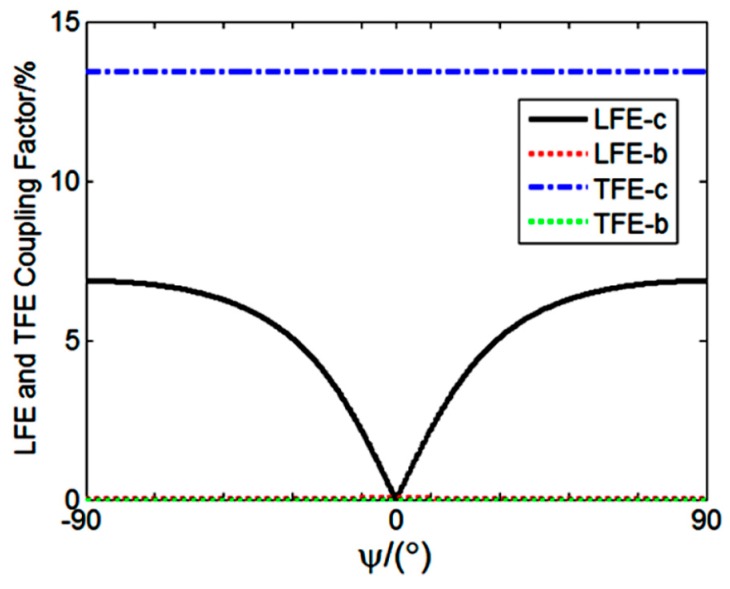
Distributions of coupling factors for lateral-field-excitation (LFE) and thickness-field-excitation (TFE) of (yxl) 13.8° LGT crystals.

The results are presented as the relationship of the piezoelectric coupling factors and the electrical field angle (ψ) in Figure 1. The electric field angle is the one between the exciting electrical field orientation and the crystallographic axis *x*. It indicates that piezoelectric coupling factors have symmetric distribution around the electrical field angle ψ = 0°. Furthermore, it is observed that the thickness-field-excitation coupling factors of the b-mode and the LFE coupling factors of the b-mode are zero. On the other hand, for zero electrical field angle (ψ = 0°), the LFE and TFE coupling factors for the c-mode reach 0 and 14.0%, respectively. Therefore, the wave for thickness-shear-mode of the c-mode can be excited for zero electrical field angle. Studies show that the thickness field is the effective one for the pseudo-LFE mode. Therefore, the (yxl) 13.8° LGT device operates in the pseudo-LFE mode when ψ = 0°. Figure 1 indicates that the device has a 14.0% piezoelectric coupling factor for the TFE c-mode. This is about two times higher than the one for the AT-cut quartz.

Equation (1) presents piezoelectrically stiffened Christoffel equation [13].

(1) $$\begin{array}{l} k^{2}\left(l_{iK}\left\{c_{KL}^{E}+\frac{\left(e_{Kj}m_{j}\right)\left(n_{i}f_{iL}\right)}{n_{i}\varepsilon_{ij}^{S}m_{j}}\right\}r_{Lj}\right)v_{j}=\rho \omega^{2}v_{i},\\ \left(i;\;j=1,2,3;\;K;L=1,2,3,4,5,6\right) \end{array}$$

where k, $l_{iK}$, $m_{j}$, $n_{i}$, $r_{Lj}$ are the wave vector, the wave propagation orientation matrix, the electric field orientation matrix, the transposed matrix of $m_{j}$, and the transposed matrix of $l_{iK}$, respectively. Moreover, $c_{KL}^{E}$, $e_{Kj}$ and $f_{iL}$ denote the stiffness matrix for a fixed electric field, the piezoelectric stress matrix and the transposed matrix of $e_{Kj}$, respectively. Furthermore,$\varepsilon_{ij}^{S}$, ρ, $v_{i}$ and ω are the permittivity constant matrix for a fixed strain, the material density, the acoustic wave phase speed, and the angle frequency, respectively. By using Equation (1), the phase speed *Va* for the TFE-c mode is calculated to be 2884.3 m/s for (yxl) 13.8° LGT crystals.

The TSM resonance frequency of a standing wave between two surfaces of a device is defined as the following:

(2) $$f_{s}=\frac{V_{a}}{2d}$$

Equation (2) shows that the plate thickness d can be determined by using the demanded frequency $f_{s}$ and the wave phase speed $V_{a}$.

## 3. Experimental

The (yxl) 13.8° LGT crystal is processed to 0.2902 mm thickness, while the corresponding resonance frequency is 4.969 MHz according to Equation (2).

**Figure 2:**
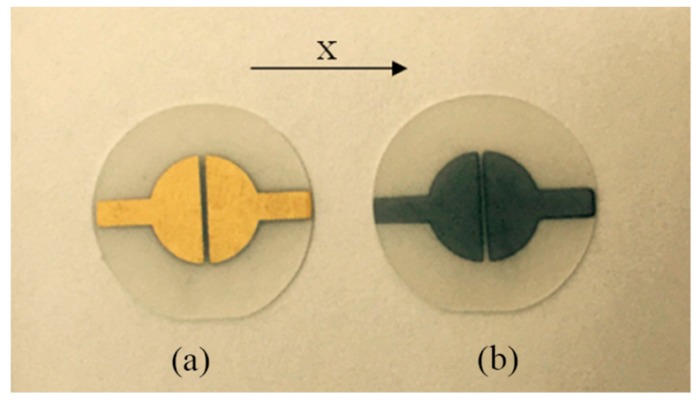
The photos of the (yxl) 13.8° Langatate (LGT) LFE device: (**a**) the side with electrode, (**b**) the side with no electrode.

Figure 2 shows the fabricated LFE device, which is designed using the (yxl) 13.8° LGT plate. Devices consist of a thin circular crystal disk with a diameter of 25.4 mm. The disk has two electrodes on one side, while there is no electrode on the other side. Electrodes are semi-circled, where the electrode diameter and gap between electrodes are set to 12.7 mm and 1mm, respectively. In order to reach ψ = 0°, the gap orientation must be set normal to the crystallographic axis *x*. Moreover, the vacuum evaporation is employed to fabricate electrodes. It should be indicated that electrodes consist of chromium and gold layers with 2 nm and 200 nm thicknesses respectively.

In order to prepare a sensing cell, a Crystal holder (CHC-100, INFICON Co. Ltd, Bad Ragaz, Switzerland) is applied to clamp the device in the present work. This holder can clamp the crystal and make only the free side of plate (without electrodes) keep contacting the liquid. A rubber seal ring and the tubular structure of the holder make the surface with electrodes stay in a closed space. Thus, the surface with electrodes cannot contact with liquid when the sensor is used in liquid phase sensing. Furthermore, an Agilent 4395A impedance analyzer is employed to validate the cut of pseudo LFE on LGT crystals. It is intended to test the electrical impedance distributions of the (yxl) 13.8° LGT LFE devices in air and water mediums.

The sensor holder is immersed into the experiment medium. In this section, it is intended to investigate the device sensitivity to the variation of the medium conductivity and permittivity by using NaCl and 2-propanol aqueous solutions, respectively. The concentrations are set to 0–0.03 wt% and 0–90 wt% for NaCl and 2-propanol solutions, respectively. Moreover, sugarcane aqueous solutions with concentrations varying within 0–8 wt% are employed to investigate the device performances in a variety of acoustic viscosities. The acoustic viscosity (AV) for a medium is defined as the scalar multiplication of the aqueous solution viscosity and the density. Moreover, the frequency-time responses of devices in different concentrations of 2-propanol aqueous solutions are investigated. In this regard, a FC-1300 frequency counter and an SRS phase-locked oscillator are applied to investigate the frequency responses. It should be indicated that the experiments are carried out at room temperature.

## 4. Results and discussion

### 4.1. Investigation of the Impedance Properties

**Figure 3:**
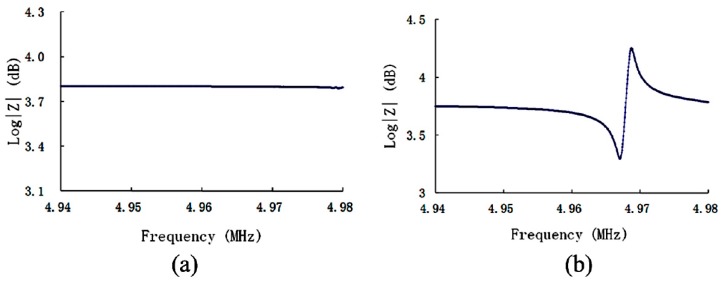
Impedance properties of the (yxl) 13.8° LGT LFE device in different mediums: (**a**) air (**b**) water.

Figure 3a illustrates the impedance properties for the (yxl) 13.8° LGT LFE device when air is used as the medium. It indicates that the impedance is almost constant, with no abrupt change. On the other hand, Figure 3b shows a significant peak in the impedance properties of the device in the water medium. Figure 3b indicates that the frequency corresponding to the resonance peak is 4.967 MHz. The observed peak for the water medium may be interpreted in the following way: The water performs as a virtual electrode and forms two serial TFEs, which leads to the above-described phenomena. The observed resonance frequency is slightly lower than that of the TFE-c (i.e., slow-shear) mode, namely, 4.969 MHz. This discrepancy may be attributed to the impact of impedances of the medium loading. Therefore, the observed peak in resonance is interpreted as the TFE-c mode. Moreover, the effective electric field of the pseudo-LFE mode is the TFE electric field. Hence, it is concluded that the (yxl) 13.8° LGT LFE device performs on the pseudo-LFE mode, which is in consistency with the theoretical predictions of Section 2. 

### 4.2. Measurement of Sensing Characteristics

#### 4.2.1. Measurement of the Liquid Conductivity

**Figure 4:**
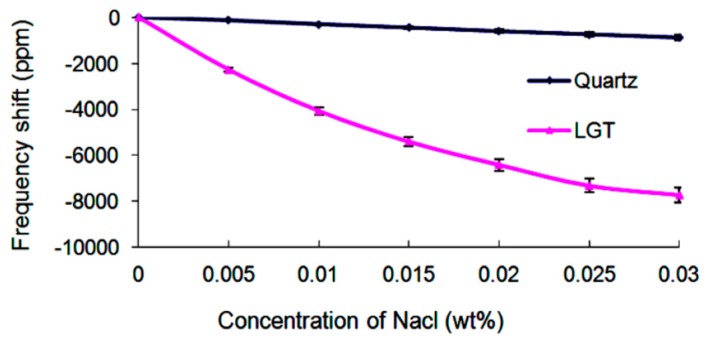
Frequency changes of different LFE devices for different concentrations of NaCl aqueous solutions.

For the NaCl solutions with different concentrations considered in the present study, specifically for the concentrations varying from 0 to 0.03 wt%, the relative permittivity is almost constant and shows negligible variations of 0.3 [14]. Such small variation leads to 30 ppm frequency variation for the LGT LFE device. Moreover, Hu et al. [1] showed that variations of viscosity and density can be ignored. Therefore, it is concluded that the variations of the frequency are mainly originated from the variation of the medium conductivity. Figure 4 illustrates the frequency changes of different devices for the variety of NaCl concentrations. When the experiment is carried out on the AT-cut quartz device, the resonance frequency change is only 855 ppm as the NaCl solution concentration varies from 0 to 0.03 wt%. On the other hand, (yxl) 13.8° LGT LFE device displays high sensitivity to the variation of the medium conductivity and has a resonance frequency variation of 7722 ppm. This is approximately 9 times higher than that of the AT-cut quartz device.

#### 4.2.2. Measurements of the Liquid Permittivity

**Figure 5:**
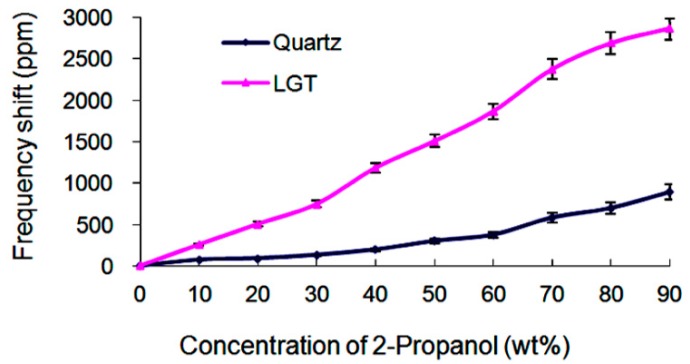
Frequency changes of different LFE devices for different concentrations of 2-propanol aqueous solutions.

In this section, the aqueous solution concentration of the 2-propanol varies from 0 to 90 wt%. The conductivity is almost constant and shows minimal variations from 0 to 2.5 × 10^−4^ S/m [15]. This variation leads to 28 ppm variation in the frequency of the LGT LFE device. Furthermore, the scalar multiplication of the viscosity and density varies from 0 to 0.105 cp.g/mL [16]. For the LGT LFE device, this yields a frequency variation smaller than 48 ppm. Therefore, frequency variations are mainly originated from the medium permittivity variations. Figure 5 illustrates the variations of frequency of different devices for a variety of 2-propanol concentrations. It indicates that as the solution concentration varies within 0–90 wt%, the frequency variation of the LGT device is approximately 2871 ppm, while such a variation for the AT-cut quartz is 897 ppm. It is concluded that the sensitivity of the LGT LFE device is approximately 3.2 times higher than that of the quartz LFE device for the variation of the medium permittivity.

#### 4.2.3. Measurements of the Liquid Acoustic Viscosity

**Figure 6:**
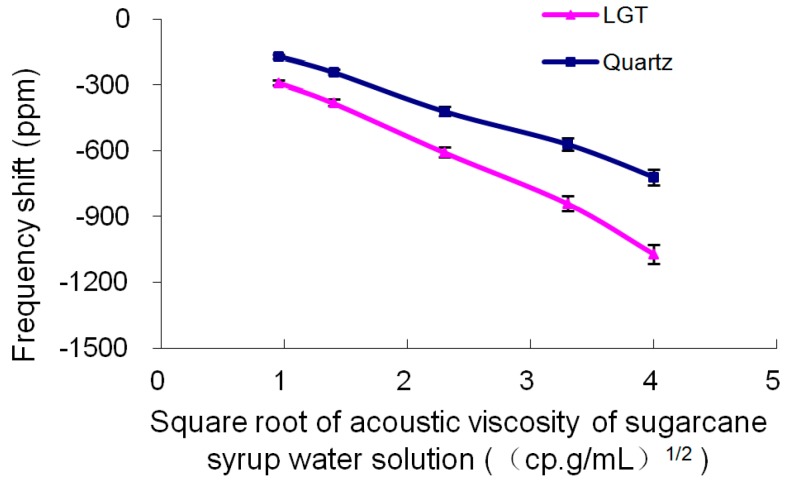
Frequency changes of different LFE devices for a variety of acoustic viscosities of sugarcane aqueous solutions.

For sugarcane aqueous solutions with different concentrations varying from 0 to 8 wt%, the variations of the corresponding acoustic viscosity is from 1 to 16 cp.g/mL, respectively. On the other hand, the relative permittivity is almost constant and shows minimal variations from 0 to 0.4 [17]. These fluctuations lead to a small frequency variation (i.e., less than 28 ppm) for the LGT LFE device. Moreover, variations of the conductivity can be ignored. Therefore, variations of the frequency are mainly attributed from the variations of the medium acoustic viscosity. Figure 6 shows the frequency variations of different devices for a variety of acoustic viscosities of sugarcane aqueous solutions. It is observed that for the LGT LFE device and varying square root of the acoustic viscosities from 1 to 4 (cp.g/mL)^1/2^, the variation of the LGT LFE resonance frequency is approximately 776 ppm, while such variation for the resonance frequency of the quartz LFE device is 551 ppm. It is concluded that the sensitivity of the LGT LFE devices to variations of the acoustic viscosity is about 1.4 times higher than the one for quartz LFE devices. 

### 4.3. Measurements of the Frequency-Time Responses

**Figure 7:**
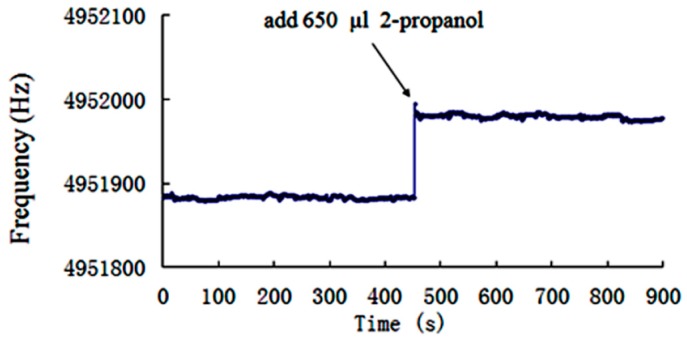
Frequency time responses of the LGT LFE device in aqueous solutions of 2-Propanol with a minor concentration variation (the initial concentration is 60%).

**Figure 8:**
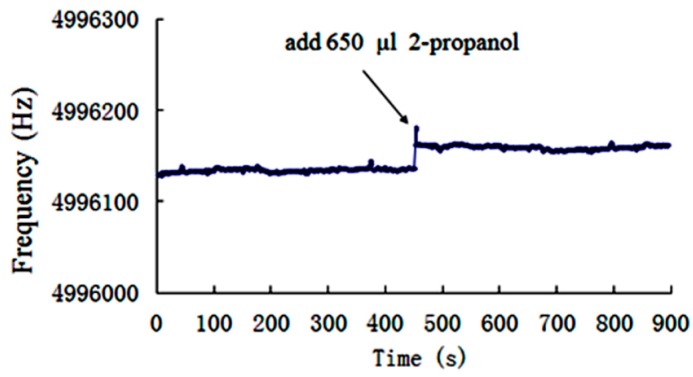
Frequency time responses of the quartz LFE device in aqueous solutions of 2-Propanol with a minor concentration variation (the initial concentration is 60%).

During the experiment carried out in the present study, devices are first immersed into 150 mL of 60% 2-Propanol aqueous solutions. Then, 650 μL 2-Propanol is added to the solution after 450 s and the mixture is completely stirred by means of a magnetic mixer. The solution concentration is increased by 0.17% and the permittivity is decreased by 0.405. The frequency-time responses of LGT and quartz LFE devices are measured and the results are illustrated in Figure 7 and Figure 8, respectively. It is observed the LGT LFE device showed larger frequency shifts. This can be attributed to its higher sensitivity to the medium permittivity. Furthermore, output frequencies of devices increased abruptly, when the solution concentration increases slightly, and then tend to be stable within 3 s. Moreover, Figure 7 and Figure 8 show that after 453 s, the maximum frequency fluctuating of two devices are both approximately ±6 Hz. It is concluded that two devices have the similar frequency stabilities.

In the above experiments, the (yxl) 13.8° LGT LFE device exhibits higher sensitivity to variations of the conductivity, relative permittivity and acoustic viscosity of the aqueous solution in comparison to the AT-cut quartz LFE device. For pseudo–LFE devices in gaseous medium, no peak is observed in the resonance distribution. On the one hand, LFE coupling factors are all zero (shown in Figure 1). And yet, TFE coupling factors cannot take effect because there is no electrode on the sensing surface. For the aqueous medium, when the sensing surface of the device contacts with the aqueous medium, the adjacent liquid can interact with the electric field by its level of conductivity and relative permittivity, so that the electric field can be influenced. As a consequence of the constraints in the electrical boundary conditions at the sensing surface, the redistribution of the electric field from the lateral toward the thickness direction occurs. As a result, TFE coupling factors can take effect with the liquid acting as a virtual electrode. Furthermore, for a device with higher piezoelectric coupling factor, the device resonance parameters face the larger variations originating from the variations in the aqueous solution characteristics because of the piezoelectric coupling effect. It is concluded that the higher sensitivity of the (yxl) 13.8° LGT LFE device to the variation of the aqueous solution characteristics may be attributed to a higher piezoelectric coupling factor. The piezoelectric coupling factor of (yxl) 13.8° LGT is almost 2 times greater than the one for the AT–cut quartz.

## 5. Conclusions

In this work, pseudo-LFE acoustic wave characteristics of LGT crystals and the according LFE sensors were investigated. The present study shows that the (yxl) 13.8° LGT plate operates on the pseudo-LFE mode, when it was excited by the lateral field with the direction parallel to the crystallographic axis x of the piezoelectric plate. Furthermore, the (yxl) 13.8° LGT sensor was designed and manufactured. Devices were investigated from an impedance property and sensitivity point of view. The results were compared to the ones from the AT-cut quartz LFE devices. The results indicate that compared to the AT-cut quartz LFE sensor, the (yxl) 13.8° LGT sensor had 9 and 3.2 times higher sensitivity to the variations of aqueous solution conductivity and permittivity, respectively. The liquid under study must be strongly conducting. Also, it was found that the (yxl) 13.8° LGT sensor had higher sensitivities to variations of the liquid acoustic viscosity, in comparison to the AT-cut quartz LFE device. In studying interfacial phenomena and sensing, it is necessary to test the variations of the mechanical and electrical characteristics of analytes. Therefore, the (yxl) 13.8° LGT LFE sensor may be a good choice in liquid phase sensing applications.
